# C-Reactive Protein is Associated with Brain White Matter Anomalies in Gulf War Illness

**DOI:** 10.29245/2572.942x/2020/3.1276

**Published:** 2020-10-01

**Authors:** Peka Christova, Lisa M. James, Adam F. Carpenter, Scott M. Lewis, Rachel A. Johnson, Brian E. Engdahl, Apostolos P. Georgopoulos

**Affiliations:** 1 Brain Sciences Center, Department of Veterans Affairs Health Care System, Minneapolis, MN, 55417, USA; 2 Department of Neuroscience, University of Minnesota Medical School, Minneapolis, MN 55455, USA; 3 Department of Psychiatry, University of Minnesota Medical School, Minneapolis, MN 55455, USA; 4 Department of Neurology, University of Minnesota Medical School, Minneapolis, MN 55455, USA; 5 Department of Psychology, University of Minnesota Medical School, Minneapolis, MN 55455, USA

**Keywords:** Gulf War Illness, C-Reactive Protein, Diffusion Tensor Imaging

## Abstract

Independent lines of research have documented elevated peripheral inflammation and brain white matter alterations in Gulf War Illness (GWI). We recently documented an association of C-reactive protein (CRP), a marker of inflammation, and decreased fornix white matter integrity in GWI. The aim of the present study was to extend those findings to evaluate the association between CRP and white matter anisotropy and diffusion throughout the brain in GWI. Sixty-three veterans with GWI provided blood samples for evaluation of CRP and underwent a 3T magnetic resonance imaging scan from which fractional anisotropy (FA), axial diffusivity (AD), radial diffusivity (RD), and mean diffusivity (MD) were obtained. An additional index characterizing the shape of the diffusion ellipsoid, Ca, which reflects deviation from sphericity (or isotropy) was obtained. Results demonstrated that CRP was significantly associated with decreased FA and Ca and with increased RD and MD, but not AD. These findings documenting a highly significant association between peripheral inflammation and specific white matter alterations in GWI are discussed in terms of GWI-related exposures that may promote systemic inflammation and deleterious neural effects downstream.

## Introduction

Gulf War Illness (GWI) is increasingly recognized as a neuroimmune condition characterized by both neurological alterations and immune system disruptions hypothesized to stem from 1990–1991 Gulf War service-related exposures in genetically vulnerable individuals^1,2^. To that end, evidence of immune dysfunction including elevated peripheral inflammatory markers such as C-reactive protein (CRP) have been reported in veterans with GWI^3–6^ as have numerous structural and functional brain abnormalities involving both gray matter and white matter^7–11^. Furthermore, recent studies have documented associations between elevated CRP and brain abnormalities in veterans with GWI including evidence of cortical thinning and hippocampal atrophy^10,11^. With the exception of one study that documented an inverse association between CRP and fractional anisotropy of the fornix, the major output of the hippocampus^10^, the association of CRP with white matter integrity in GWI is unknown.

White matter anatomy is commonly evaluated with diffusion tensor imaging (DTI) which can be used to measure the movement of water molecules within axons^12,13^. Under normal conditions, water molecules are constrained by axonal membranes to diffuse mainly along the long axis of the axon; however, the loss of axonal integrity alters diffusion properties. The most common DTI measures – fractional anisotropy (FA), axial diffusivity (AD), radial diffusivity (RD), and mean diffusivity (MD) - are extensively discussed elsewhere^12–17^. Briefly, FA reflects the extent to which diffusion is restricted. Values range from 0 to 1 where lower values indicate less restriction (by axonal membranes), or isotropy, and higher values suggest that diffusion is anisotropic, or occurs in only one direction. FA is inversely related to directional diffusivity measures. These include AD, which describes diffusivity in the direction of the principal axis of the fiber tract, RD, which describes diffusion perpendicular to the primary axis, and MD, which is the combination of AD and RD. To describe specifically the shape of the diffusion tensor, additional metrics including linear (Cl), planar (Cp), and spherical (Cs) measures are used that represent its prolate, oblate, and spherical components, respectively. From these measures, Ca, which reflects deviation from sphericity, can be derived^18^.

Diffusion tensor imaging (DTI) studies have documented several white matter alterations in veterans with GWI. These include increased mean diffusivity in the right superior longitudinal fasciculus and increased axial diffusivity, particularly involving the right inferior fronto-occipital fasciculus, in veterans with GWI relative to civilian controls^19^. A study comparing Gulf War veterans exposed to sarin and cyclosarin relative to unexposed veterans found evidence of increased radial diffusivity and pervasive axial diffusivity in the exposed veterans^20^. Significantly lower FA and higher RD and MD were reported in Gulf War veterans with chronic pain compared to healthy control Gulf War veterans^21^. Finally, a recent study documented decreased FA was inversely associated with CRP in Gulf War veterans^10^. In the present study we extended those findings by examining the association of C-reactive protein to several measures of brain white matter anisotropy and diffusivity in Gulf War veterans.

## Materials and Methods

### Participants

Sixty-three GWI patients (59 men and 4 women) were studied after providing an informed consent in accordance with the Declaration of Helsinki. GWI status was determined using a self-report symptom checklist that permits classification as GWI case or control according to the Center for Disease Control^22^ and the Kansas criteria^23^. The Center for Disease Control definition requires one or more symptoms in at least two domains that include fatigue, pain, or mood and cognition. The more restrictive Kansas criteria requires that veterans report moderate to severe symptoms in at least 3 of 6 domains: fatigue, pain, neurological/cognitive/mood, skin, gastrointestinal, and respiratory. All GWI veterans in the present study met both case definitions. Consistent with the Kansas criteria case definition, veterans were excluded from the study if they reported medical or psychiatric conditions that could account for GWI symptoms or impair reporting^2^. Individuals with traumatic brain injury were also excluded from the study. The study was approved by the University of Minnesota and Minneapolis VA Health Care System Institutional Review Boards.

### Body Mass Index (BMI)

BMI was computed using the height and weight of the participant (BMI = kg/m^2^).

### CRP

Non-fasting peripheral venous blood samples were collected for evaluation of high sensitivity C- reactive protein and analyzed using standard procedures by the Minneapolis VAHCS Clinical Laboratory.

### Magnetic Resonance Imaging (MRI) Acquisition

All data were acquired using a Philips 3.0 T MR scanner (Achieva, Philips Healthcare, Best, The Netherlands). In the initial phase of the study, data were acquired from 18 participants using a phased array SENSitivity Encoding (SENSE) 8-channel head coil for reception. For each participant a high resolution T1-weighted Turbo Field Echo (T1w TFE SENSE) was obtained (168 sagittal slices, TR = 8.1932 ms, TE =3.7520 ms, Acquisition matrix 240 × 240, Flip angle 8 deg., voxel size 0.9375 mm × 0.9375 mm × 1 mm). Subsequently, upgrades were applied to the system and data were acquired for the remaining 45 participants using a phased array SENSitivity Encoding (SENSE) 15-channel head coil for reception. For each participant a high resolution T1-weighted Turbo Field Echo (T1w TFE SENSE) was obtained (168 sagittal slices, TR =8.0928 ms, TE = 3.698 ms, Acquisition matrix 240 × 240, Flip angle 8 deg., voxel size 0.7500 mm × 0.7500 mm × 1 mm).

Diffusion weighted images (DWI, DTI_medium_iso_E) consisted of a single-shot echo-planar imaging sequence (EPI, TR=11.023 s, TE = 55 ms, Acquisition matrix 112 × 112, 70 slices with 2 mm thickness without gap, in-plane resolution 0.875 mm × 0.875 mm). Images were acquired in the axial plane with diffusion gradients applied in 15 non-collinear directions with a b-value of 1000 s/mm^2^ and one non-diffusion weighted image with a b-value of 0 s/mm^2^. In advance of each acquisition a capsule of Vitamin E was taped to the participant’s right temple to determine orientation in the imaged data.

## Image Processing

Diffusion data were analyzed using the default parameter settings in the diffusion MR toolbox Explore DTI, version 4.8.6 (www.exploredti.com^24^). Anatomical T1-w images for each participant were linearly transformed into Montreal Neurological Institute (MNI) 152 space using AFNI (Analysis of Functional NeuroImages, afni.nimh.nih.gov). DTI Images were corrected for head movement and eddy current induced geometric distortions using the procedure described in Leemans and Jones^25^ and corrected for EPI/susceptibility distortion^26^. Then the image was warped to an ‘undistorted’ T1-w modality, in the MNI-152 template using the ELASTIX approach^27^ with non-rigid registration. The above steps were applied to the data obtained from each participant and done in one interpolation to reduce blurring effects, resulting in streamline files. The quality of each processing step (tensor estimation, T1-w transformation) was visually inspected. The valid co-registration was checked by overlaying the respective images for each participant.

Explore DTI provided the Mori standard labeled atlas in the same MNI 152 standard space (ICBM-DTI-81^28,29^). By warping the atlas template and transforming the associate labels to each individual data set, diffusion metrics were calculated in each of the atlas areas. A total of 48 white matter areas were identified excluding brainstem and cerebellar regions. Global areas are: Middle cerebellar peduncle, Pontine crossing tract, Genu, Body and Splenium of corpus callosum, Fornix (column and body of fornix). Bilateral regions included following atlas labels from both hemispheres: Corticospinal tract, Medial lemniscus, Inferior and Superior cerebellar peduncle, Cerebral peduncle, Anterior limb of the internal capsule, Posterior limb of the internal capsule, Retrolenticular part of the internal capsule, Anterior, Superior and Posterior corona radiata, Posterior thalamic radiation, Sagittal stratum, External capsule, Cingulum (cingulate gyrus), Cingulum (hippocampus), Fornix (cres) Stria terminalis, Superior longitudinal fasciculus, Superior fronto-occipital fasciculus, Uncinate fasciculus, and Tapetum.

DTI measures

For each brain region in the white matter atlas, the following metrics were obtained.

Fractional anisotropy (FA):

(1) $$FA=\sqrt{\frac{3}{2}}\frac{\sqrt{{\left(\lambda_{1}-\overset{ˆ}{\lambda }\right)}^{2}+{\left(\lambda_{2}-\overset{ˆ}{\lambda }\right)}^{2}+{\left(\lambda_{3}-\overset{ˆ}{\lambda }\right)}^{2}}}{\sqrt{\lambda_{1}^{2}+\lambda_{2}^{2}+\lambda_{3}^{2}}}$$

Where $\overset{ˆ}{\lambda }=\left(\lambda_{1}+\lambda_{2}+\lambda_{3}\right)/3$ is the mean of the eigenvalues of the diffusion tensor.

Mean diffusivity (MD):

(2) $$MD=\left(\lambda_{1}+\lambda_{2}+\lambda_{3}\right)/3$$

Axial Diffusivity (AD):

(3) $$AD=\lambda_{1}$$

Radial Diffusivity (RD):

(4) $$RD=\left(\lambda_{1}+\lambda_{2}\right)/2$$

We also computed an anisotropy measure ($C_{a}$) to capture the deviation from the spherical case, as follows. First, we sorted the eigenvalues of the diffusion vector such that $\lambda_{1}\geq \lambda_{2}\geq \lambda_{3}\geq 0$ and then computed

(5) $$C_{a}=\frac{\lambda_{1}\;+\;\lambda_{2}\;-\;2\lambda_{3}}{\lambda_{1}\;+\;\lambda_{2}\;+\;\lambda_{3}}$$

## Data analysis

For each brain, 48 white matter ROIs x 5 measures (FA, MD, AD, RD, $C_{a}$) = 240 values were available, for a total of 63 brains × 240 values/brain = 15120 data values; namely, 3024 values per measure above. Standard statistical methods were used to analyze the data, including descriptive statistics (mean, SEM) and partial correlations. The two conditions of data acquisition were coded as a binary variable (8 channel head coil = 0; 15 channel head coil = 1) and used as a controlled variable (with age) in calculating the partial correlation between CRP and DTI measure. All statistical analyses were done using the IBM-SPSS statistical package (versions 23 and 26).

## Results

### Descriptive statistics

#### Age.

**Figure 1.**
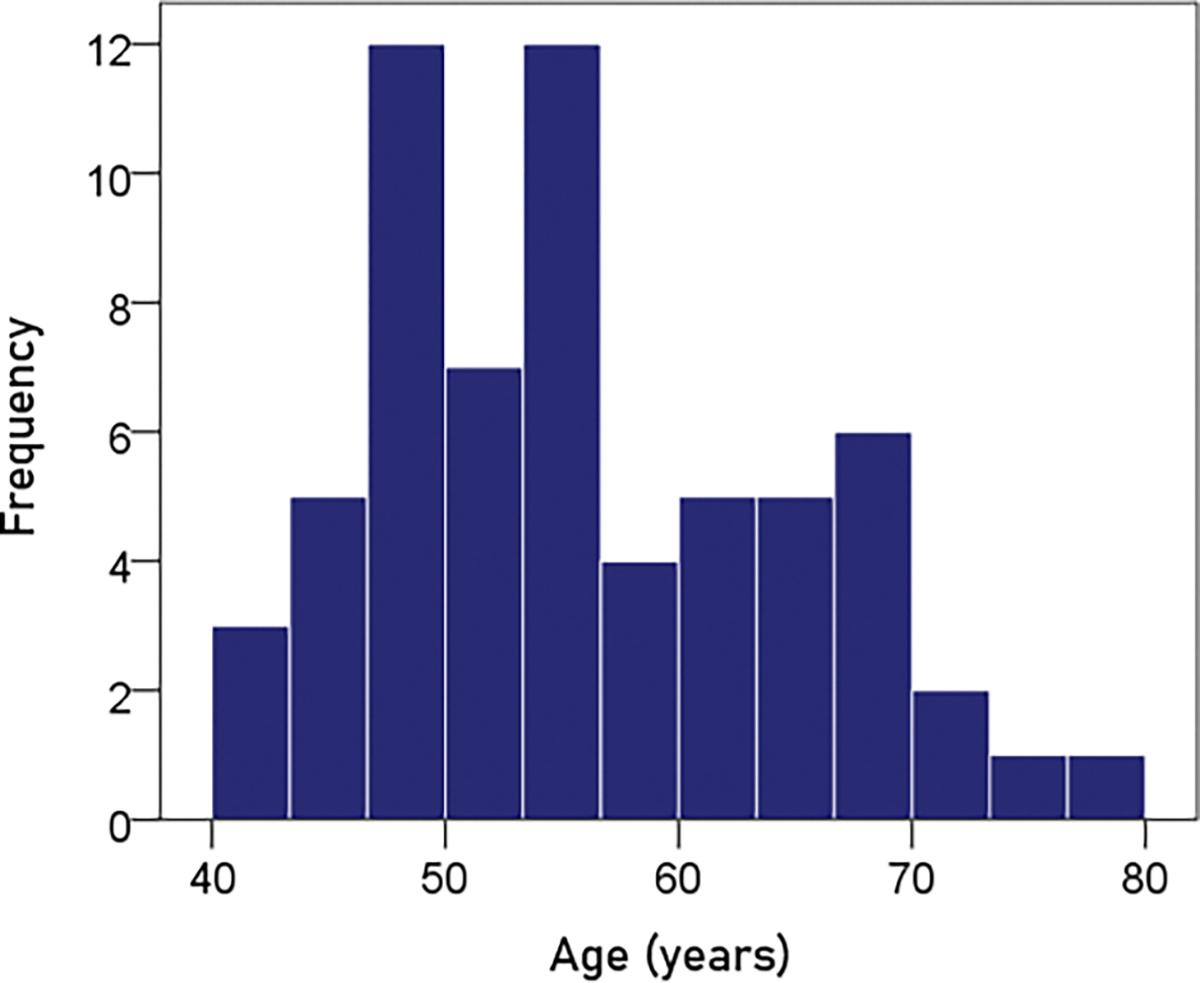
Frequency distribution of the age of the participants (N = 63).

The frequency distribution of age is shown in Figure 1; the mean ± SEM age was 55.2 ± 1.12 y.

#### BMI.

**Figure 2.**
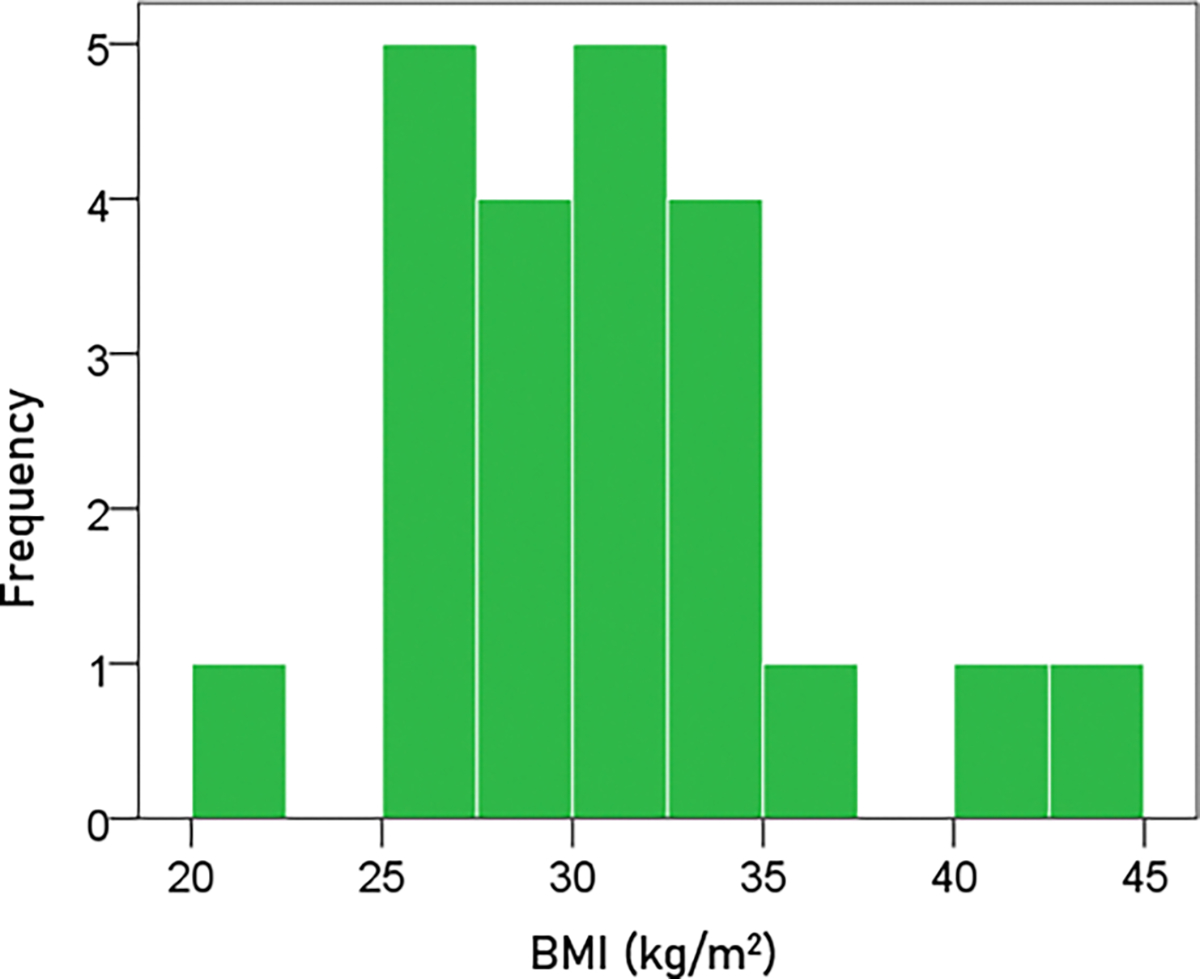
Frequency distribution of the BMI of the participants (N = 63).

The frequency distribution of BMI is shown in Figure 2. The mean ± SEM BMI was 31.3 ± 0.64 kg/m^2^ (N = 63).

#### CRP.

**Figure 3.**
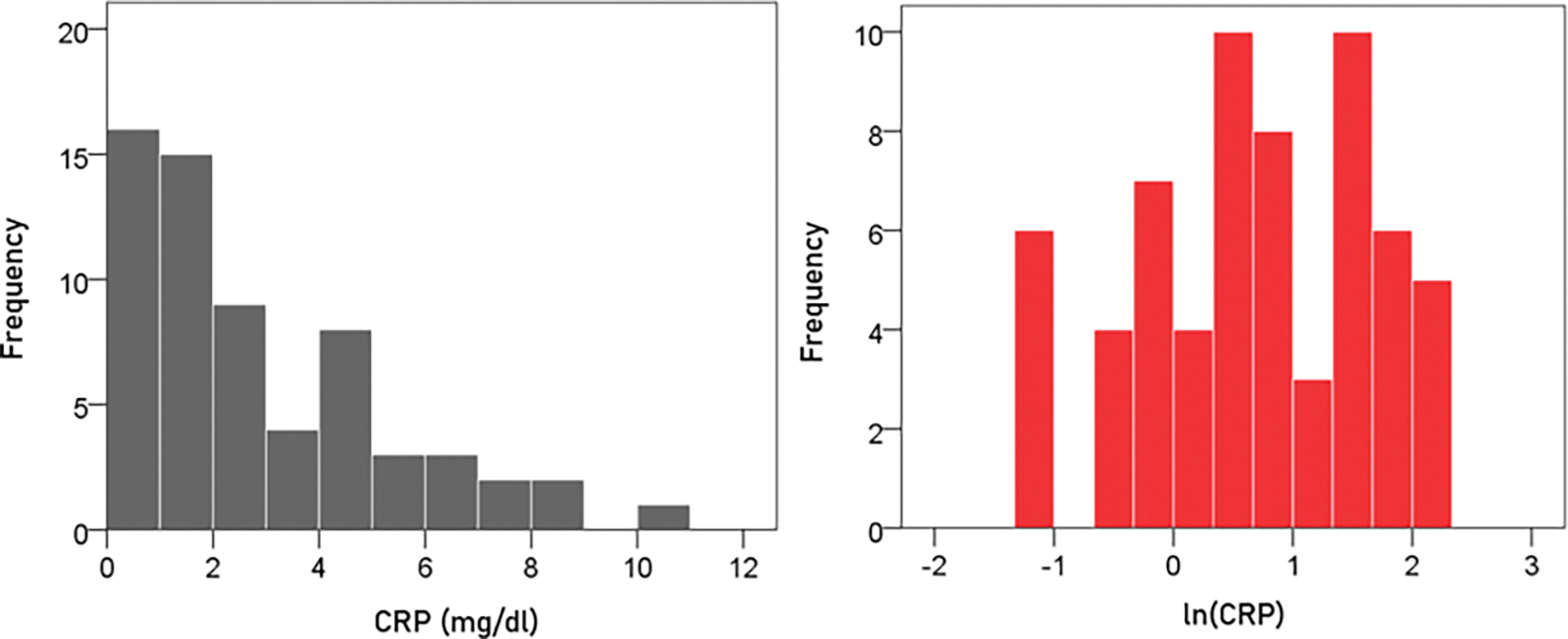
Frequency distribution of the CRP of the participants (left panel) and its natural-log transformed values (right panel; N = 63). See text for details.

The frequency distribution of CRP values (mg/dl) is shown in the left panel of Figure 3. It can be seen that it is skewed to the right, suggesting a logarithmic transformation. Indeed, a natural-log transformation made the distribution more symmetric (Fig. 3, right panel).


(6) $$ln(CRP)={log}_{e}(CRP)=ln(CRP)$$

A detailed analysis of this distribution showed the absence of outliers. The mean ± SEM of ln(CRP) was 0.674 ± 0.122 (N = 63).

#### *FA* (Figure 4).

The mean ± SEM of FA were 0.436 ± 0.0015674 (N = 3024).

#### MD.

The mean ± SEM of MD were 0.0009163 ± 0.000005148 (N = 3024).

#### AD.

The mean ± SEM of AD were 0.001347 ± 0.000006168 (N = 3024).

#### RD.

The mean ± SEM of RD were 0.0007007 ± 0.000004848 (N = 3024).

#### $C_{a}$.

The mean ± SEM of $C_{a}$ were 0.5802 ± 0.0015272818 (N = 3024).

### Association between age and DTI measures

Age was significantly associated with all DTI parameters and, therefore, was retained as a covariate in further analyses. The respective Pearson correlation coefficients were as follows; P values were Bonferroni-adjusted for 5 multiple comparisons. N = 3014 for all.


$$Age\;and\;FA.r=-0.141\left(P=3.28\times 10^{-14}\right).$$

$$Age\;and\;MD.r=0.096\left(P=5.87\times 10^{-7}\right).$$

$$Age\;and\;AD.\;r=0.062\left(P=0.003\right).$$

$$Age\;and\;RD.\;r=0.114\left(P=1.55\times 10^{-9}\right).$$

$$Age\;and\;C_{a}.r=-0.141\left(P=4.01\times 10^{-14}\right).$$

### Association between BMI and DTI measures

BMI was not significantly associated with any DTI measure (P > 0.1 for all) and, therefore, was not retained as a covariate in further analyses.

### Association between gender and DTI measures

Gender was not significantly associated with any DTI measure (P > 0.7 for all) and, therefore, was not retained as a covariate in further analyses.

### Association between ln(CRP) and DTI measures

The association between ln(CRP) and DTI measures was evaluated by calculating the partial correlation between them, controlling for age. P values below for the ln(CRP) effect were Bonferroni-corrected for 5 multiple comparisons. ln(CRP) was significantly associated with all DTI parameters except for *AD*. The partial correlations $r_{p}$ (controlling for age) were as follows. N = 3024 for all.


$$\begin{array}{l} ln\left(CRP\right)\;\text{and}\;\text{FA.}\;r_{p}=-0.059\left(P=0.001\right).\\ ln\left(CRP\right)\;\text{and}\;\text{MD.}\;r_{p}=0.057\left(P=0.002\right).\\ ln\left(CRP\right)\;\text{and}\;RD.r_{p}=0.063\left(P=0.001\right).\\ ln\left(CRP\right)\;\text{and}\;C_{a}.r_{p}=-0.058\left(P=0.001\right). \end{array}$$

The same values of $r_{p}$ were obtained when the binary variable coding for 8- and 15-channel coil acquisitions was added (with age) and a controlled variable.

**Figure 5.**
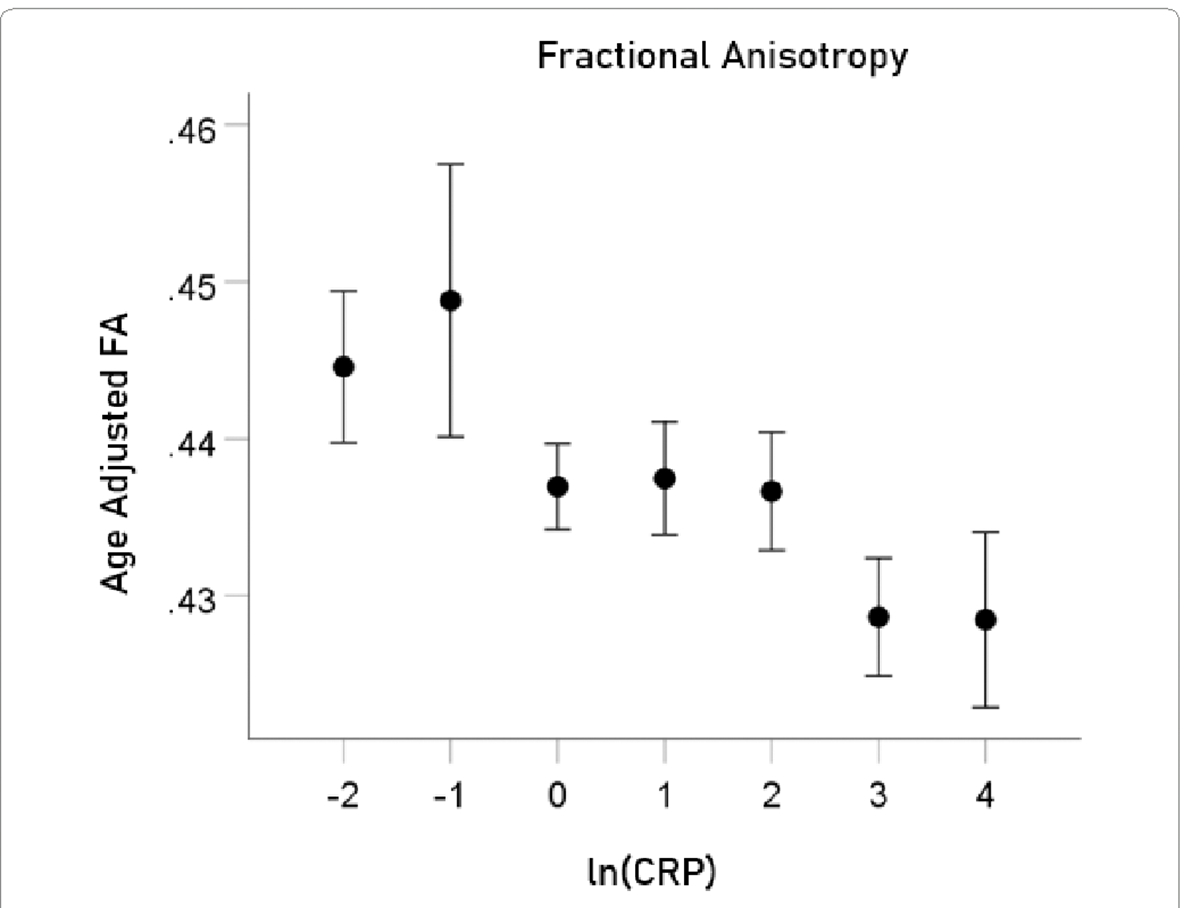
Age-adjusted FA values (mean ± SEM) are plotted against binned ln(CRP); bin width = 1. See text for details.

**Figure 6.**
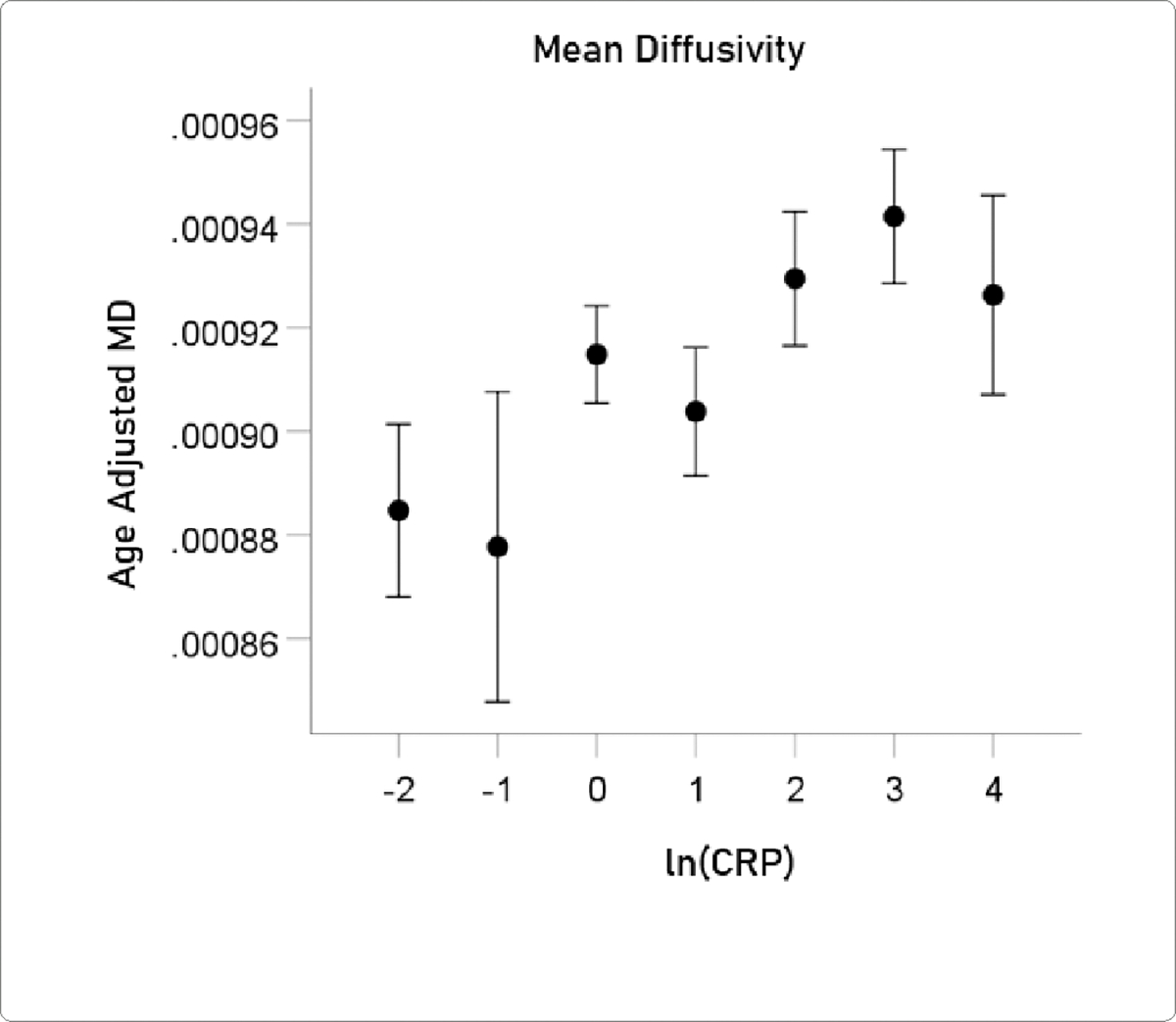
Age-adjusted MD values (mean ± SEM) are plotted against binned ln(CRP); bin width = 1. See text for details.

**Figure 7.**
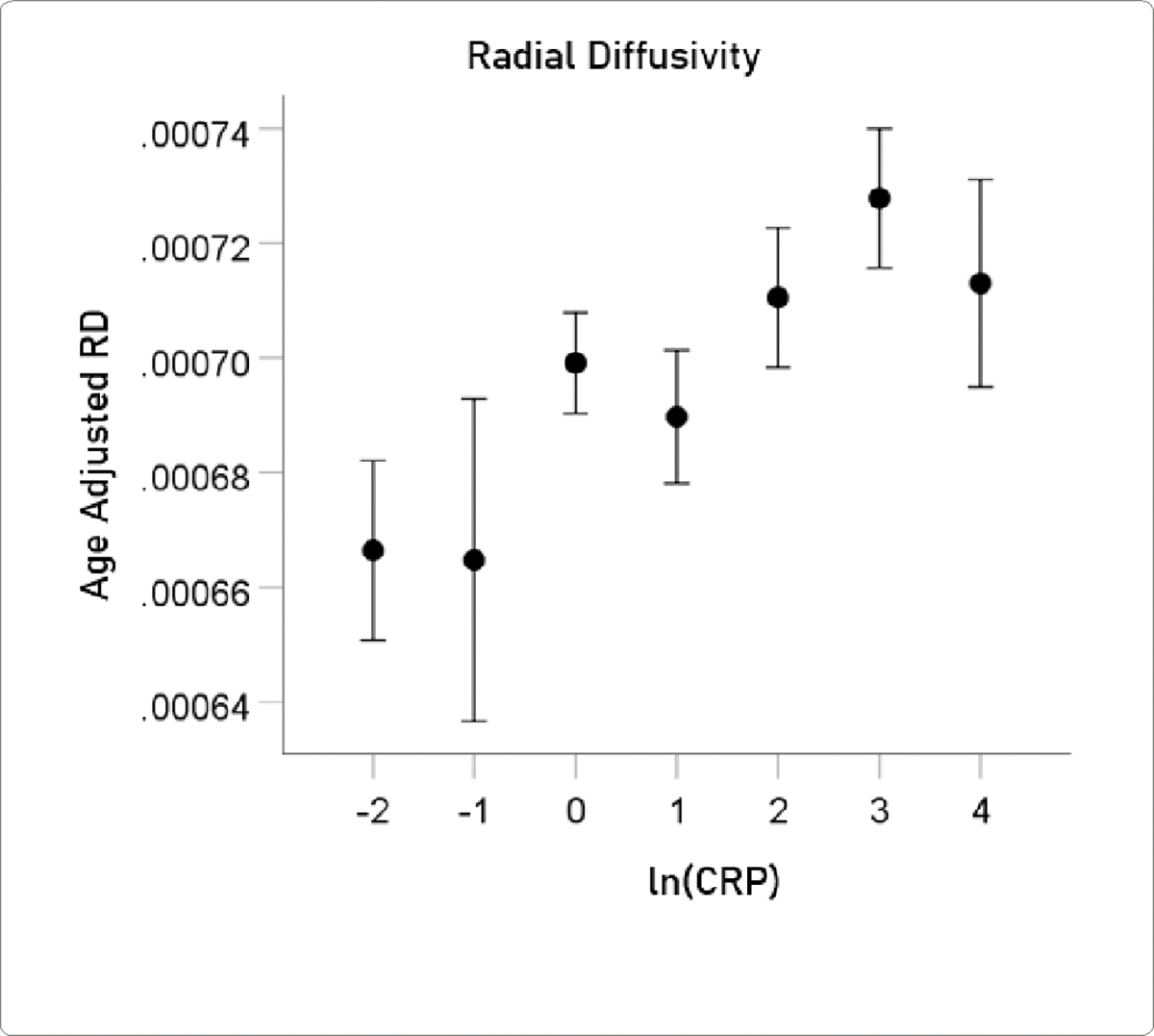
Age-adjusted RD values (mean ± SEM) are plotted against binned ln(CRP); bin width = 1. See text for details

**Figure 8.**
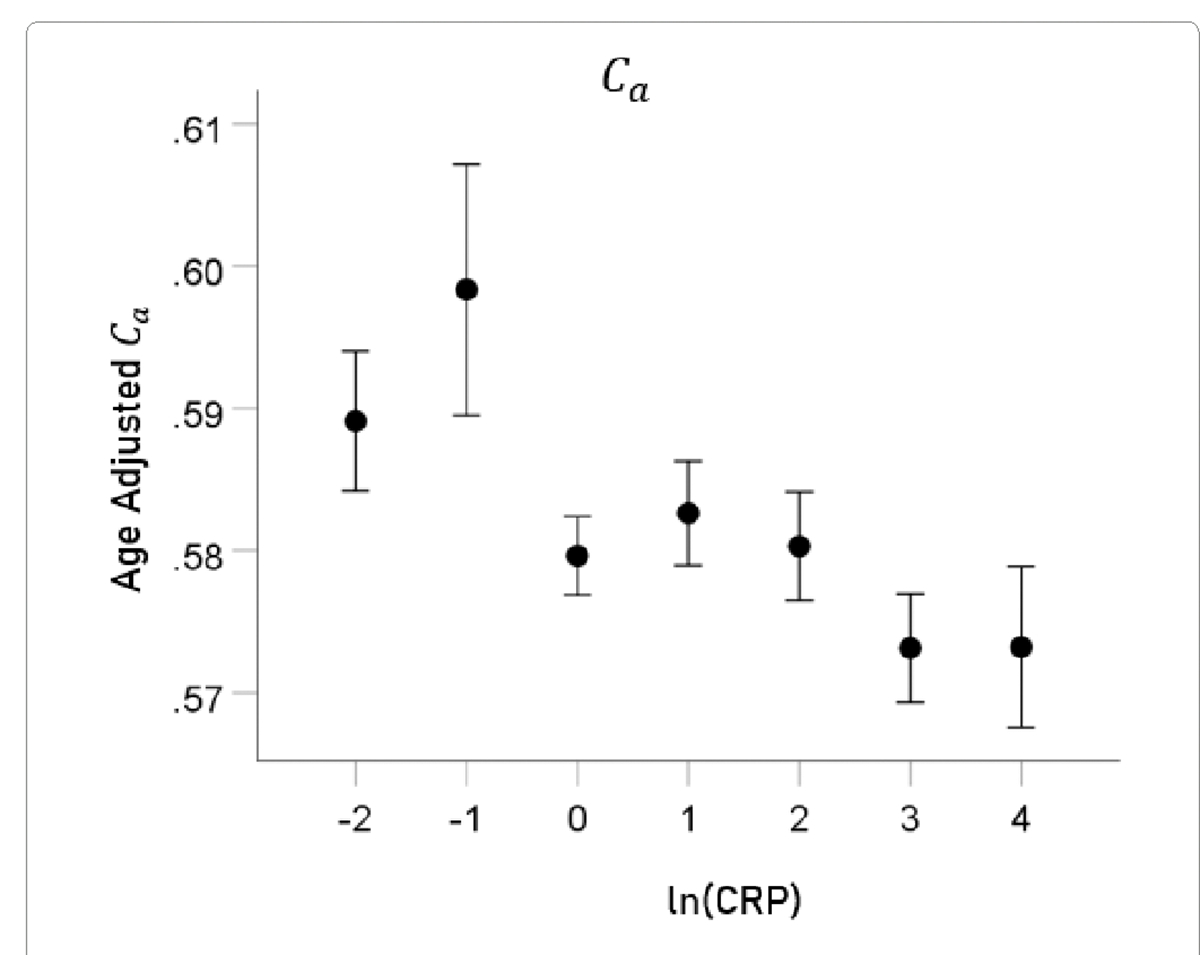
Age-adjusted $C_{a}$ values (mean ± SEM) are plotted against binned ln(CRP); bin width = 1. See text for details.

For visualization purposes, the relations above are illustrated in Figures. 5–8 as plots of age-adjusted DTI measures against binned ln(CRP) values with bin width = 1.

## Discussion

Here we investigated the association of a peripheral marker of inflammation, C-reactive protein, with white matter anisotropy and diffusivity in veterans with GWI. Findings from the present study revealed highly significant associations between CRP and white matter alterations including decreased FA and Ca and increased RD and MD, but not AD, in Gulf War veterans. The results provide further evidence of brain anomalies associated with GWI and document an association of GWI-related brain effects and inflammation.

Previous studies have documented associations between peripheral inflammation and white matter alterations^30,31^. Here we extend that to GWI and show that most, but not all, DTI metrics we evaluated were associated with peripheral inflammation in GWI. This dissociation of findings is informative as the different DTI metrics reflect different processes. Indeed, the use of multiple DTI measures is recommended to optimally characterize white matter microstructure^16^. For instance, FA is a highly sensitive yet non-specific indicator of white matter microstructural changes, the nature of which can be clarified by considering other DTI metrics such as AD and RD. AD and RD are considered to be indicators of axonal degeneration and demyelination, respectively^32^. Here, we reported an association of inflammation and RD, but not AD, suggesting an effect of inflammation on demyelination, but not axonal degeneration, in GWI. That is not to say that axonal degeneration is not associated with GWI; in fact, prior studies have documented increased AD in GWI^20,21^. Rather, the AD effect does not appear to be associated with inflammation. On the other hand, demyelination and inflammation are intimately linked as exemplified most definitively by multiple sclerosis^33^. We previously demonstrated that magnetoencephalography-based brain communication in GWI is indistinguishable from well-established immune-related disorders including relapsing-remitting multiple sclerosis, Sjogren’s syndrome, and rheumatoid arthritis, yet highly different from healthy controls and psychiatric disorders^1^. The current findings suggest that white matter alterations including myelin damage may contribute to the observed similarities in neural communication. To that end, autoantibodies to myelin proteins^34^ and oligodendrocyte impairment^35^ have been implicated in GWI, further supporting a role of demyelination in GWI.

Though the brain was historically considered immune-privileged, substantial evidence suggests that CRP and other inflammatory molecules can access the brain through several routes and influence local inflammatory pathways^36–38^. The source of systemic inflammation in GWI is uncertain; however, evidence increasingly points to pathogen exposure in veterans lacking specific immunity against those pathogens in contributing to GWI^39–45^. We have speculated that lack of immunogenetic protection against pathogens results in their persistence, and consequently promotes inflammation, autoimmunity, and ultimately neural damage^40,46^. In vitro studies have identified specific pathogens circulating in serum of veterans with GWI that contribute to harmful effects on neural cell cultures; remarkably, those damaging effects are ameliorated with the concomitant addition of antibodies^41–44^, suggesting potential avenues for intervention in vivo.

### Limitations

There are two methodological limitations in this study. The first limitation concerns the low number of directions (N = 15) in DTI data acquisition. Although future studies with higher directional resolution should provide more precise DTI measurements, we believe that the associations between CRP and DTI measures observed here will hold. The second limitation concerns the possible influence of white matter hyperintensities (WMHs) on DTI measures^47,48^. Although we did not quantify WMHs, their presence/absence was similar to that of the general population, as evaluated by reviewing the scans by Dr. Carpenter (coauthor of this paper and an expert in multiple sclerosis). As stated by Svärd et al.^48^, “if the purpose of a study is to compare alterations in NAWM between two groups using DTI it may be necessary to adjust the statistical analysis for WMH.” (abstract in ref.^48^)In this study, we did not compare two groups and, therefore, given the qualitative assessment of WHM presence above, we feel that our results regrading CRP are valid. Nevertheless, this issue remains to be investigated in future studies.

## Figures and Tables

**Figure 4.**
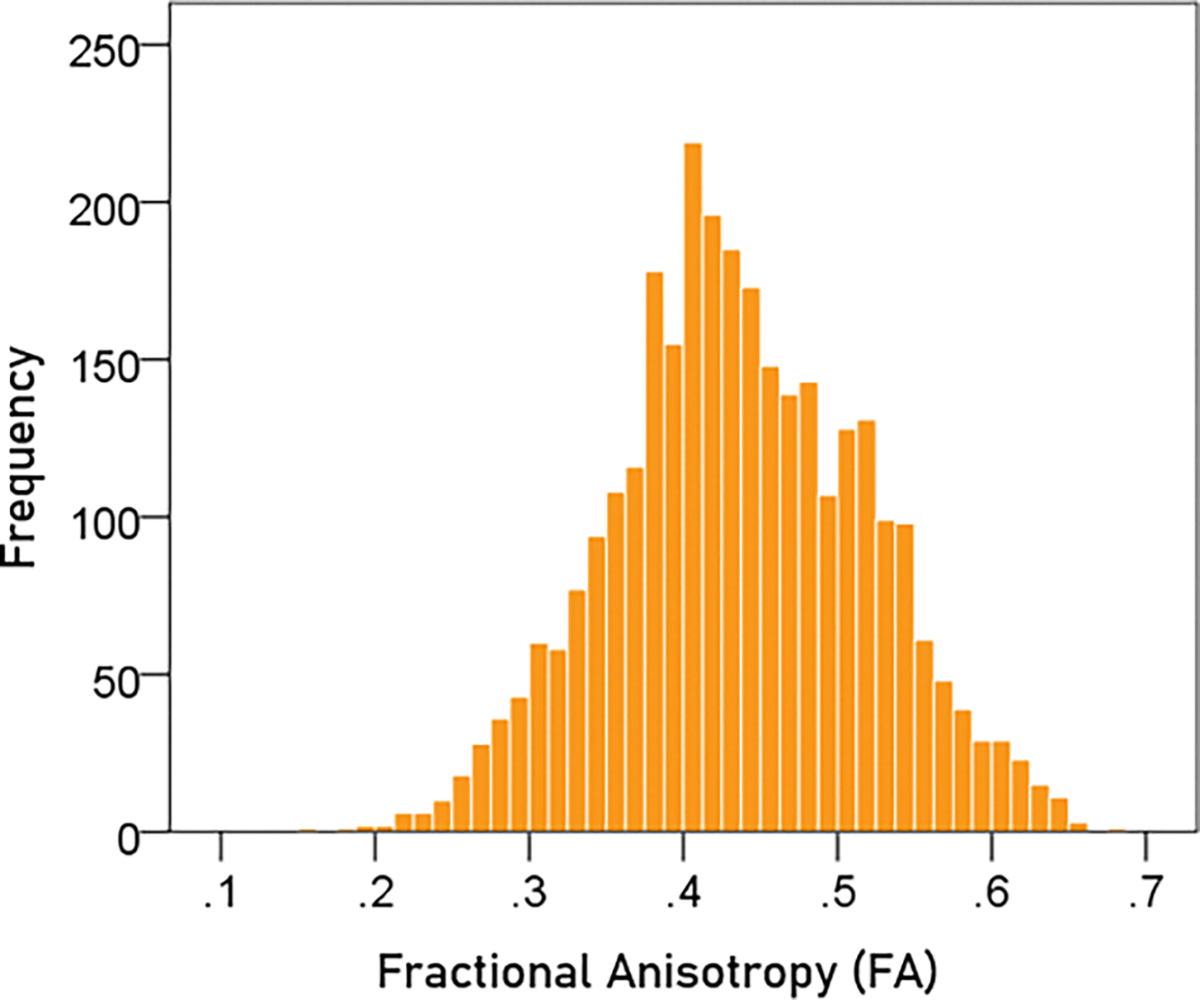
Frequency distribution of FA (N = 3024). See text for details.

## References

[1] Georgopoulos AP, James LM, Carpenter AF, Gulf War illness (GWI) as a neuroimmune disease. Exp Brain Res. 2017; 235(10): 3217–3225. 28762055 10.1007/s00221-017-5050-0

[2] Coughlin SS. A neuroimmune model of Gulf War Illness. J Environ Health Sci. 2017; 3: 10.15436/2378-6841.17.1665. PMC5976257 29862319

[3] James LA, Engdahl BE, Johnson RA, Gulf War Illness and Inflammation: Association of symptom severity with C-reactive protein. J Neurol Neuromed. 2019; 4(2): 15–19. 10.29245/2572.942x/2019/2.1245 PMC12311971 40746955

[4] Johnson GJ, Leis LA, Slater BC, Elevated platelet count, C-reactive protein and thromboxane analog-induced platelet aggregation in patients with Gulf War veterans’ illnesses: evidence of a chronic inflammatory state? Blood Coagul Fibrinolysis. 2013; 24: 736–741. 23751609 10.1097/MBC.0b013e328362627f

[5] Johnson GJ, Slater BC, Leis LA, Blood biomarkers of chronic inflammation in Gulf War Illness. PLoS One. 2016; 11(6): e0157855 27352030 10.1371/journal.pone.0157855 PMC4924830

[6] Kelsall HL, McKenzie DP, Sim MR, Physical, psychological, and functional comorbidities of multisymptom illness in Australian male veterans of the 1991 Gulf War. Am J Epidemiol. 2009; 170: 1048–1056. 19762370 10.1093/aje/kwp238

[7] White RF, Steele L, O’Callaghan JP, Recent research on Gulf War illness and other health problems in veterans of the 1991 Gulf War: effects of toxicant exposures during deployment. Cortex. 2016; 74: 449–475. 10.1016/j.cortex.2015.08.022 PMC4724528 26493934

[8] Engdahl BE, James LM, Miller RD, A magnetoencephalographic (MEG) study of Gulf War Illness (GWI). EBioMedicine. 2016; 12: 127–32. 27592598 10.1016/j.ebiom.2016.08.030 PMC5078573

[9] Christova P, James LM, Engdahl BE, Subcortical brain atrophy in Gulf War Illness. Exp Brain Res. 2017; 235: 2777–2786. 28634886 10.1007/s00221-017-5010-8

[10] Christova P, James LM, Carpenter AF, (in press). Gulf War Illness: C-reactive protein is associated with reduction of the volume of hippocampus and decreased fractional anisotropy of the fornix. J Neurol Neuromed. 10.29245/2572.942x/2020/3.1272 PMC12077250 40371006

[11] Christova P, James LM, Carpenter AF, Lewis SM, (under review). Human Leukocyte Antigen (HLA) alleles prevent metabolically-induced inflammation and cerebrocortical thinning in Gulf War Illness. 10.29245/2572.942x/2020/3.1273 PMC12077253 40371004

[12] Basser PJ, Mattiello J, LeBihan D. MR diffusion tensor spectroscopy and imaging. Biophys J. 1994; 66(1): 259–67. 8130344 10.1016/S0006-3495(94)80775-1 PMC1275686

[13] Basser PJ, Mattiello J, LeBihan D. Estimation of the effective self-diffusion tensor from the NMR spin echo. J Magn Reson. 1994; 103: 247–254. 10.1006/jmrb.1994.1037 8019776

[14] Le Bihan D, Breton E, Lallemand D, MR imaging of intravoxel incoherent motions: application to diffusion and perfusion in neurologic disorders. Radiology. 1986; 161(2): 401–7. 3763909 10.1148/radiology.161.2.3763909

[15] Le Bihan D Molecular diffusion nuclear magnetic resonance imaging. Magn Reson Q. 1991; 7(1): 1–30. 2043461

[16] Alexander AL, Lee JE, Lazar M, Diffusion tensor imaging of the brain. Neurotherapeutics. 2007; 4(3): 316–29. 17599699 10.1016/j.nurt.2007.05.011 PMC2041910

[17] Assaf Y, Pasternak O. Diffusion Tensor Imaging (DTI)-based White Matter Mapping in Brain Research: A Review. J Mol Neurosci. 2008; 34: 51–61. 18157658 10.1007/s12031-007-0029-0

[18] Alexander AL, Hasan K, Kindlmann G, A geometric analysis of diffusion tensor measurements of the human brain. Magn Reson Med. 2000; 44: 283–291. 10918328 10.1002/1522-2594(200008)44:2<283::aid-mrm16>3.0.co;2-v

[19] Rayhan RU, Stevens BW, Raksit MP, Exercise challenge in Gulf War Illness reveals two subgroups with altered brain structure and function. PLoS One. 2013; 8(6): e63903. 23798990 10.1371/journal.pone.0063903 PMC3683000

[20] Chao LL, Zhang Y, Buckley S. Effects of low-level sarin and cyclosarin exposure on white matter integrity in Gulf War Veterans. Neurotoxicology. 2015; 48: 239–48. 25929683 10.1016/j.neuro.2015.04.005 PMC5493444

[21] Van Riper SM, Alexander AL, Koltyn KF, Cerebral white matter structure is disrupted in Gulf War Veterans with chronic musculoskeletal pain. Pain. 2017; 158(12): 2364–2375. 28796115 10.1097/j.pain.0000000000001038

[22] Fukuda K, Nisenbaum R, Stewart G, Chronic multisymptom illness affecting Air Force veterans of the Gulf War. JAMA. 1998; 280: 981–988. 9749480 10.1001/jama.280.11.981

[23] Prevalence Steele L. and patterns of Gulf War illness in Kansas veterans: association of symptoms with characteristics of person, place, and time of military service. Am J Epidemiol. 2000; 152: 992–1002. 11092441 10.1093/aje/152.10.992

[24] Leemans A, Jeurissen B, Sijbers J, ExploreDTI: a graphical toolbox for processing, analyzing, and visualizing diffusion MR data. Proc. 17th Sci Meeting of Intl Soc Mag Reson Med 2009; Honolulu, USA: p.3537

[25] Leemans A, Jones DK. The B-matrix must be rotated when correcting for subject motion in DTI data. Magn Reson Med. 2009; 61(6): 1336–49. 19319973 10.1002/mrm.21890

[26] Irfanoglu MO, Walker L, Sarlls J, Effects of image distortions originating from susceptibility variations and concomitant fields on diffusion MRI tractography results. NeuroImage. 2012; 61(1): 275–88. 22401760 10.1016/j.neuroimage.2012.02.054 PMC3653420

[27] Klein S, Staring M, Murphy K, Elastix: A toolbox for intensity-based medical image registration. IEEE Trans Med Imaging. 2010; 29(1): 196–205. 19923044 10.1109/TMI.2009.2035616

[28] Mori S, Oishi K, Jiang H, Stereotaxic white matter atlas based on diffusion tensor imaging in an ICBM template. NeuroImage. 2008; 40(2): 570–82. 18255316 10.1016/j.neuroimage.2007.12.035 PMC2478641

[29] Mori S, Wakana S, Zijl PCM van, MRI Atlas of Human White Matter. Elsevier; 2005.

[30] Walker KA, Windham BG, Power MC, The association of mid-to late-life systemic inflammation with white matter structure in older adults: The Atherosclerosis Risk in Communities Study. Neurobiol Aging. 2018; 68: 26–33. 29702373 10.1016/j.neurobiolaging.2018.03.031 PMC6010227

[31] Wersching H, Duning T, Lohmann H, Serum C-reactive protein is linked to cerebral microstructural integrity and cognitive function. Neurology. 2010; 74(3): 1022–1029. 20350977 10.1212/WNL.0b013e3181d7b45b

[32] Winklewski PJ, Sabisz A, Naumczyk P, Understanding the physiopathology behind axial and radial diffusivity changes—what do we know? Front Neurol. 2018; 9: 92. 29535676 10.3389/fneur.2018.00092 PMC5835085

[33] Stadelmann C, Wegner C, Brück W. Inflammation, demyelination, and degeneration—recent insights from MS pathology. Biochimica et Biophysica Acta (BBA)-Molecular Basis of Disease. 2011; 1812(2): 275–282. 20637864 10.1016/j.bbadis.2010.07.007

[34] Brahmajothi MV, Abou-Donia MB. Autoantibodies to myelin proteins as biomarkers to identify nervous system injuries in Gulf War Illness and other neuromuscular disorders. J Immunol. 2018; 200(Supplement 1): 16662.

[35] Belgrad J, Dutta DJ, Bromley-Coolidge S, Oligodendrocyte involvement in gulf war illness. Glia. 2019; 67(11): 2107–24. 31339622 10.1002/glia.23668 PMC6899710

[36] Elwood E, Lim Z, Naveed H, The effect of systemic inflammation on human brain barrier function. Brain Behav Immun. 2017; 62: 35–40. 27810376 10.1016/j.bbi.2016.10.020 PMC5380128

[37] Quan N, Banks WA. Brain-immune communication pathways. Brain Behav Immun. 2007; 21(6): 727–735 17604598 10.1016/j.bbi.2007.05.005

[38] D’Mello C, Le T, Swain MG. Cerebral microglia recruit monocytes into the brain in response to tumor necrosis factor alpha signaling during peripheral organ inflammation. J Neurosci. 2009; 29(7): 2089–2102. 19228962 10.1523/JNEUROSCI.3567-08.2009 PMC6666330

[39] Georgopoulos AP, James LM, Mahan MY, Reduced Human Leukocyte Antigen (HLA) protection in Gulf War Illness (GWI). EBioMedicine. 2016; 3: 79–85 26870819 10.1016/j.ebiom.2015.11.037 PMC4739436

[40] James LM, Christova P, Engdahl BE, Human leukocyte antigen (HLA) and Gulf War Illness (GWI): HLA-DRB1*13:02 spares subcortical atrophy in Gulf War veterans. EBioMedicine. 2017; 26: 126–131. 29137891 10.1016/j.ebiom.2017.11.005 PMC5832612

[41] Tsilibary EPC, Souto EP, Kratzke M, Anthrax and Gulf War Illness (GWI): Evidence for the Presence of Harmful Anthrax Antigen PA63 in the Serum of Veterans with GWI. J Neurol Neuromed. 2019; 4(6): 1–9. 10.29245/2572.942x/2019/6.1255 PMC12311968 40747435

[42] Tsilibary EPC, Souto EP, Kratzke M, Anthrax Protective Antigen 63 (PA63): toxic effects in neural cultures and role in Gulf War Illness (GWI). Neurosci Insights; in press. 10.1177/2633105520931966 PMC7328487 32656531

[43] Georgopoulos AP, Tsilibary EP, Souto EP, Adverse effects of Gulf War Illness (GWI) serum on neural cultures and their prevention by healthy serum. J Neurol Neuromed. 2018; 3(2): 19–27. 10.29245/2572.942X/2018/2.1177 PMC6486178 31032476

[44] Tsilibary CEP, Souto EP, James LM, Human immunoglobulin G (IgG) neutralizes adverse effects of Gulf War Illness (GWI) serum in neural cultures: Paving the way to immunotherapy for GWI. J Neurol Neuromed. 2018; 3(5): 23–28. 10.29245/2572.942X/2018/5.1219 PMC6486180 31032477

[45] Charonis S, James LM, Georgopoulos AP. In silico analysis of the binding affinities of antigenic epitopes of vaccines administered to Gulf War Veterans to specific HLA Class II alleles protective for Gulf War Illness. J Neurol Neuromed. 2019; 4(5): 23–30. 10.29245/2572.942x/2019/5.1254 PMC12311970 40746427

[46] James LM, Georgopoulos AP. Persistent antigens hypothesis: the human leukocyte antigen (HLA) connection. J. Neurol. Neuromed. 2018; 3: 27–31. 10.29245/2572.942x/2018/6.1235 PMC12077249 40370508

[47] Molloy CJ, Nugent S, Bokde ALW. Alterations in diffusion measures of white matter integrity associated with healthy aging. J Gerontol A Biol Sci Med Sci. 2019; Dec 12: glz289. doi: 10.1093/gerona/glz289. 31830253

[48] Svard D, Nilsson M, The effect of white matter hyperintensities on statistical analysis of diffusion tensor imaging in cognitively healthy elderly and prodromal Alzheimer’s disease. PLoS One. 2017; 21; 12(9):e0185239. doi: 10.1371/journal.pone.0185239 28934374 PMC5608410

